# From Chromatographic Optimisation to Bioanalysis: HPLC System Comparison and HPLC–QTRAP–MS/MS Determination of Cariprazine and Lurasidone in Human Serum, Urine, and Saliva

**DOI:** 10.3390/ijms27167090

**Published:** 2026-08-07

**Authors:** Karol Wróblewski, Anna Petruczynik, Zuzanna Rząd, Hanna Karakuła-Juchnowicz

**Affiliations:** 1 Laboratory of Commercial and Non-Commercial Clinical Trials, University of Rzeszów, Kopisto 2a, 35-959 Rzeszów, Poland; 2 Laboratory of Metabolic Processes and Chromatographic Analyses, Natural and Medical Center for Innovative Research, University of Rzeszów, Kopisto 2a, 35-959 Rzeszów, Poland; 3 Department of Inorganic Chemistry, Medical University of Lublin, Chodźki 4a, 20-093 Lublin, Poland; 4 Department of Clinical Neuropsychiatry, Medical University of Lublin, Głuska 2, 20-439 Lublin, Poland; rzadzuzanna@gmail.com (Z.R.); hanna.karakula-juchnowicz@umlub.pl (H.K.-J.)

**Keywords:** cariprazine, lurasidone, HPLC-DAD, HPLC-QTRAP-MS/MS, biological samples, chromatographic system optimisation, drug determination

## Abstract

Cariprazine (CAR) and lurasidone (LUR) are antipsychotic drugs used to treat schizophrenia. These drugs are relatively new in clinical practice, and there is a need to optimise and develop analytical methods for their determination in various biological matrices for biomedical analysis. To date, the detection of these drugs has been performed in serum and urine, but there are no methods for determining these drugs in saliva. In the first part of this study, various chromatographic systems were compared using high-performance liquid chromatography with diode array detection (HPLC-DAD) or coupled with quadrupole–linear ion trap tandem mass spectrometry (HPLC-QTRAP-MS/MS), taking into account the retention of tested compounds, system efficiency and peak symmetry. Next, a simple, rapid, and sensitive HPLC-QTRAP-MS/MS method has been developed for the determination of CAR and LUR in human serum, urine, and, for the first time, in saliva samples. Solid-phase extraction (SPE) was used for sample pre-treatment. Quantifications were carried out using a Polar RP column with a mobile phase consisting of acetonitrile and a formate buffer at pH 4.0 in gradient mode. The method was successfully applied for the determination of CAR and LUR in biological samples obtained from psychiatric patients. The findings suggest that saliva may be a non-invasive alternative for the quantification of free levels of investigated drugs, although further studies are required to clarify its relationship with plasma concentrations.

## 1. Introduction

CAR and LUR are novel second-generation antipsychotics approved for acute and maintenance treatment of schizophrenia (FDA, EMA). Additionally, CAR is approved for the acute treatment of manic or mixed episodes associated with bipolar I disorder in adults (FDA), and LUR is approved for the treatment of depressive episodes associated with bipolar I disorder as monotherapy and as adjunctive therapy with valproate or lithium (FDA) [1].

To date, several methods have been developed for determining CAR and LUR in biological samples and pharmaceutical preparations. Most CAR determinations have been performed by LC-MS using C18 columns and, less frequently, C8 columns. Most often, a mixture of acetonitrile, water, and formic acid was used as the mobile phase. For example, ultra-high-performance liquid chromatography coupled to a single-quadrupole mass spectrometer (UHPLC-MS) was used to determine CAR in whole blood for therapeutic drug monitoring in psychiatric patients [2]. Separation was performed on a C18 column with a mobile phase containing acetonitrile, water and 0.1% formic acid. A C18 column and a mixture of acetonitrile, methanol, 0.2 M aqueous ammonium acetate, and water (35:25:35:5, *v*/*v*/*v*/*v*) was applied for the determination of CAR in rat plasma and brain samples [3]. Samples were analysed by the HPLC-UV method, with a lower limit of quantification (LLOQ) of 20 ng/mL for plasma and 80 ng/g for the brain. Liquid chromatography coupled with tandem mass spectrometry (LC-MS/MS) was also used for the determination of CAR in rat plasma samples. The LLOQ obtained by LC-MS/MS was 0.5 ng/mL.

For the determination of CAR hydrochloride in bulk drug and pharmaceutical dosage by HPLC-DAD, a C18 column and mobile phase consisting of methanol and 0.1% trifluoroacetic acid buffer were applied [4]. The LOD and LOQ values, at 44.80 ng/mL and 135.60 ng/mL, respectively, were obtained using this procedure.

LC–MS/MS determination of low-dose third-generation antipsychotics and their metabolites in urine using an ultra-short C18 column and mobile phase consisting of methanol, water and 0.1% formic acid was described [5]. The LOD and LLOQ values equal to 0.02 and 0.5, respectively, were obtained.

LUR has also been most often determined by LC-MS using columns with a C18 phase and, less frequently, a C8 phase, and mobile phases containing a mixture of acetonitrile, water and formic acid. For example, determination of LUR in human plasma samples was carried out by high-performance liquid chromatography tandem mass spectrometry (HPLC–MS/MS) on a C8 column with a mobile phase containing acetonitrile, water and 0.1% formic acid [6]. The procedure was applied for pharmacokinetic investigations of LUR in patients with bipolar depression.

To investigate the interaction between LUR and olmutinib, analyte concentrations were determined using ultra-performance liquid chromatography tandem mass spectrometry (UPLC-MS/MS) [7]. Separation of analytes was performed on a UPLC C18 column with a mixture of acetonitrile, water and 0.1% formic acid. The analytes were detected by multiple reaction monitoring (MRM) in positive ion mode using a triple quadrupole tandem mass spectrometer.

LUR was also quantified in formulation by high-performance liquid chromatography (HPLC-DAD) with the use of a C18 column and a mobile phase consisting of acetonitrile, water and 0.1% formic acid [8]. Using this chromatographic system, the quantification limit was estimated at 1.40 μg/mL. In the investigations, mouse blood samples were analysed by UHPLC-MS/MS. Separation was performed on a UHPLC C18 column with a mixture of acetonitrile, water and 0.1% formic acid as the mobile phase. An electrospray ionisation source (ESI) operating in positive mode was used for detection.

A C18 column and mobile phase containing acetonitrile, water and 0.1% formic acid were also used for the determination of psychotropic drugs, including LUR, by the UPLC method [9].

Degreef et al. described a qualitative and semi-quantitative liquid chromatography–triple quadrupole and quadrupole time-of-flight mass spectrometry procedure for screening selected psychoactive drugs, including LUR in blood, for routine toxicological analyses [10]. Separation was performed on a C8 column with a mobile phase containing acetonitrile, water, and 0.1% formic acid.

The UPLC-MS/MS method was also developed for the analysis of 71 neuropsychotropic drugs, including LUR in human sera for drug concentration monitoring and toxicity screening [11]. Analytes were separated on a C18 column with a mixture of acetonitrile and water, 0.2% acetic acid and 10 mM ammonium acetate as the mobile phase. For detection, an electrospray ion source operated in both positive- and negative-ion modes with multiple ion monitoring was used.

Investigations of LUR degradation by LC-MS/MS were performed using a C18 column and a mobile phase containing methanol, water and 0.1% formic acid [12]. The LOD and LOQ obtained by the procedure were 0.091 μg/mL and 0.275 μg/mL, respectively.

Although there are methods for the determination of the investigated drugs in plasma/serum and urine, there are no methods for their quantification in saliva. The first aim of this study was to investigate the effects of different stationary and mobile phases on retention, peak symmetry, and chromatographic system efficiency for CAR and LUR. The final goal of the study was to develop an HPLC-QTRAP-MS/MS method for the determination of CAR and LUR in serum, urine, and, for the first time, saliva. The method was successfully applied for the quantification of investigated drugs in biological samples obtained from patients.

## 2. Results and Discussion

### 2.1. Comparison of Chromatographic Systems

**Figure 1:**
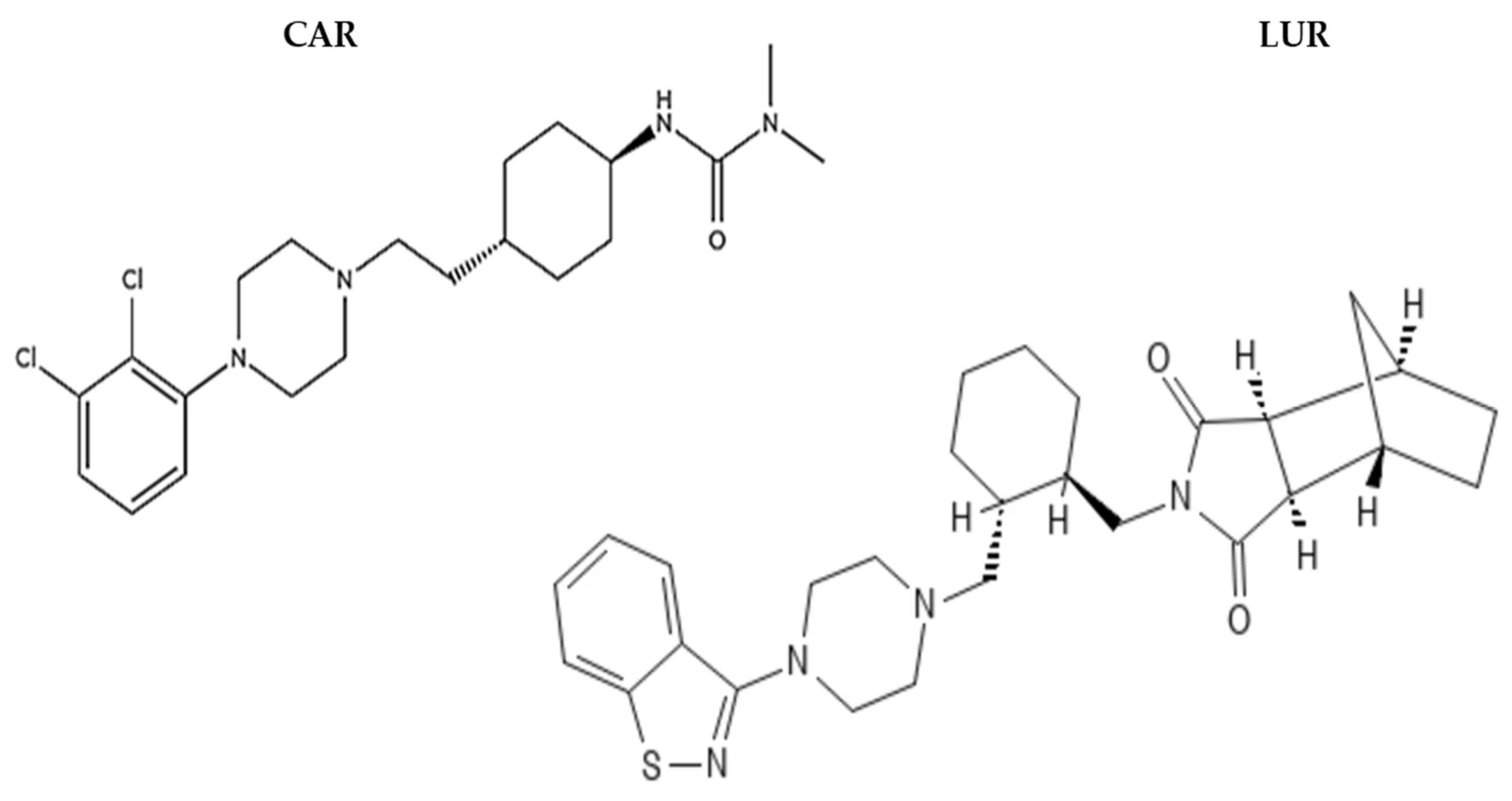
Chemical structure of CAR and LUR.

CAR and LUR standards (Figure 1) were chromatographed on Synergi Hydro-RP (Hydro RP; Phenomenex, Torrance, CA, USA), CSH Phenyl-Hexyl (Phenyl-Hexyl; Waters Corporation, Milford, MA, USA), Synergi Polar RP (Polar RP, 150 × 4.6 mm; Phenomenex, Torrance, CA, USA), ACE HILIC B (HILIC B; Advanced Chromatography Technologies, Aberdeen, UK), ACE HILIC N (HILIC N; Advanced Chromatography Technologies, Aberdeen, UK) and Luna SCX (SCX; Phenomenex, Torrance, CA, USA) columns in various eluent systems using HPLC-DAD. These chromatographic systems were compared in terms of retention of investigated psychotropic drugs on different systems that were selected according to their potential differences in retention mechanisms and, hence, the possible changes in selectivity, peak shape and performance. Most of the previously published reports on the chromatographic analysis of CAR and LUR concerned analyses performed on columns packed with the C18 stationary phase. Significant differences in retention, peak shape, and system efficiency were observed across different stationary phases. On Hydro RP, Phenyl-Hexyl and Polar RP columns, mobile phases containing 70% MeOH, 20% acetic buffer pH = 3.5, water and 0.025 mol/L DEA, 50% MeCN, 20% acetic buffer pH = 3.5, water and 0.025 mol/L DEA or 30% MeOH, 30% MeCN, 20% acetic buffer pH = 3.5, water and 0.025 mol/L DEA were tested. Across all columns and mobile-phase systems, LUR was more strongly retained than CAR. CAR and LUR were the most strongly retained on Polar RP column with mobile phase containing 70% MeOH, 20% acetic buffer pH = 3.5, water and 0.025 mol/L DEA (t_R_ values of 7.42 and 42.63 min, respectively), but the least retained on Phenyl-Hexyl with mobile phase containing 50% MeCN, 20% acetic buffer pH = 3.5, water and 0.025 mol/L DEA (t_R_ values of 2.66 and 9.99 min, respectively). On all tested HILIC columns with a mobile phase containing 90% MeCN and 0.1 M ammonium formate, both drugs were weakly retained. On HILIC A and HILIC B columns, stronger retention was obtained for CAR (t_R_ = 4.20 and 3.02 min, respectively) than for LUR (t_R_ = 2.18 and 2.01 min, respectively). Retention of both drugs on the HILIC N column was similar (for CAR, t_R_ = 2.33 min; for LUR, t_R_ = 2.48 min). In the SCX column, both investigated drugs were strongly retained despite the use of a high ionic strength and a high organic modifier concentration in the mobile phase (t_R_ > 120 min).

The results regarding peak symmetry and system efficiency obtained on different columns using different mobile phases were also examined. Great differences in peak shape were observed on different chromatographic systems. In almost all mobile-phase systems applied on Hydro RP, Phenyl-hexyl and Polar RP columns, symmetrical peaks were obtained (As values between 0.8 and 1.5). Only on the Hydro RP column with a mobile phase containing 50% MeCN, 20% acetic buffer pH = 3.5, water and 0.025 mol/L DEA, As = 1.71 for CAR was obtained. On all HILIC columns, significantly worse peak symmetry was observed for both drugs. The most symmetric peaks were obtained on the HILIC N column (As = 1.43 and 1.54 for LUR and CAR, respectively). The most symmetrical peaks were obtained on the Phenyl-Hexyl column, especially with a mobile phase containing 70% MeOH, 20% acetic buffer pH = 3.5, water and 0.025 mol/L DEA (As values of 1.03 and 1.08 for LUR and CAR, respectively).

The separation efficiency of the investigated systems was compared by theoretical plate number per meter (N/m). The lowest plate number was obtained for CAR on HILIC N column with a mixture of 90% MeCN and 0.1 M ammonium formate as the mobile phase (4350 N/m), while on a Polar RP column with a mobile phase containing 30% MeCN, 30% MeOH, 20% acetic buffer pH = 3.5, water and 0.025 mol/L DEA, the highest N/m values were achieved (N/m = 39,550). The lowest system efficiency for LUR was observed in a chromatographic system with a Hydro RP column and a mobile phase containing 50% MeCN, 20% acetic buffer (pH 3.5), water, and 0.025 mol/L DEA. The system with a Polar RP column and a mixture of 30% MeCN, 30% MeOH, 20% acetic buffer (pH 3.5), water, and 0.025 mol/L DEA was the most efficient for the analysis of LUR (N/m = 61,400). For both drugs, the highest N/m values were obtained in all tested mobile phases on the Polar RP column. The values obtained for the analysed parameters for various chromatographic systems are summarised in Table 1.

### 2.2. HPLC-QTRAP-MS/MS Method Development

After the initial optimisation of the chromatographic conditions using HPLC–DAD, the most suitable stationary phase was determined based on system efficiency, peak symmetry, and analyte resolution. To ensure continuity and transferability of chromatographic performance, the same stationary phase was used throughout method development for HPLC-MS/MS. A Polar RP column was therefore selected, although different column parameters were used (100 × 3 mm, 2.5 µm, 100 Å), suitable for fast HPLC coupled with mass spectrometry. Eluents compatible with spectrometry were assessed as mobile phases, including acetonitrile, acetonitrile containing 0.1% formic acid, and a formate buffer. Although the addition of DEA to the mobile phase provided very good results in HPLC-DAD, it was not applied as a mobile phase additive in HPLC-MS/MS due to its strong ion suppression effects, poor MS compatibility, and tendency to increase background noise and source contamination. Instead, a volatile formate buffer was used to ensure stable ionisation and optimal MS sensitivity. Consequently, the final conditions for the HPLC-MS/MS analysis were selected not only based on the chromatographic results, but also taking into account compatibility with the MS technique, ionisation efficiency and the long-term stability of the instrument.

A gradient system composed of acetonitrile and a 5 mmol formate buffer at pH 4.0 was finally selected for method development. The above mobile phase provided efficient separation, symmetrical peaks (As = 1.14 and 0.96 for CAR and LUR, respectively), high system efficiency (N/m = 5974 and 17,055 for LUR and CAR, respectively), good selectivity, and a strong detector response. The retention time (t_R_) for CAR was 1.9 min, and for LUR, it was 3.6 min. The total time required for a single analysis was 6.0 min.

**Figure 2:**
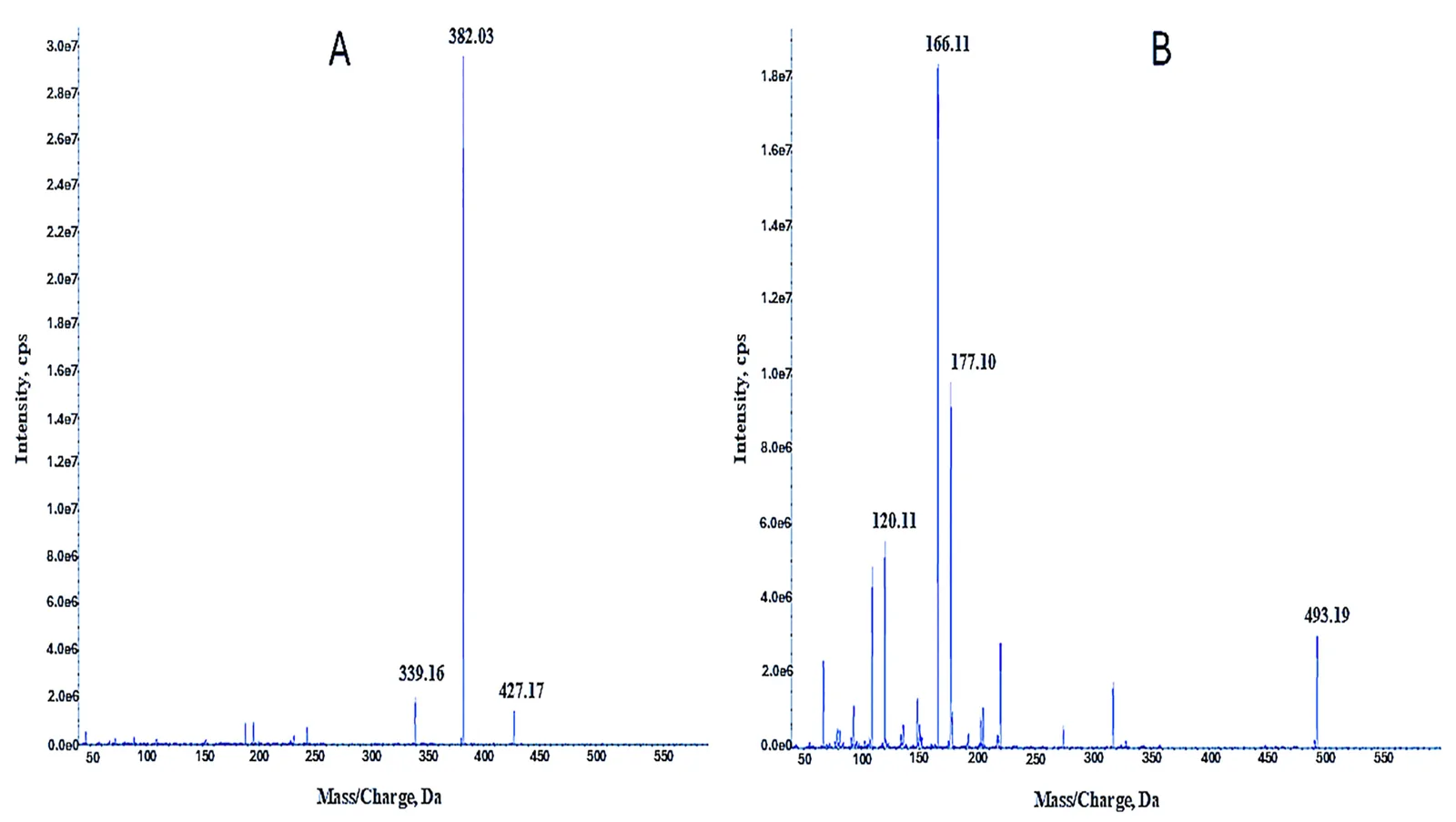
Representative MS/MS spectra obtained for CAR (**A**) and LUR (**B**). MS/MS parameters described in Table 2 were applied.

Mass spectrometry parameters (Table 2) were optimised to increase the method’s selectivity and sensitivity and to select the most intense and characteristic fragmentation ions for qualitative and quantitative analysis. MS/MS mode was used to record product ions from the real samples of each target compound. Representative MS/MS spectra of CAR and LUR are shown in Figure 2. LC-MS/MS is the most suitable for LUR and CAR analysis in biological samples due to the sensitivity of the assays.

Multiple reaction monitoring (MRM) was used for quantitative analysis. An external calibration curve was used for quantification instead of an internal standard method. Application of internal standard (IS) correction in LC-MS/MS methods for biological samples is recommended by guidelines for bioanalytical method validation. The absence of an IS should be justified [13]. Achieving satisfactory precision and accuracy without applying IS may constitute grounds for approval without its use [14]. Given the LC-MS system’s good stability, the repeatable sample preparation process, the acceptable matrix effect, and the high cost of isotopic IS, the decision was made not to incorporate an internal standard in the development of the analytical method. Using an external calibration strategy avoids the need to identify and confirm an appropriate internal standard, streamlines the analytical workflow, and reduces method complexity. The proposed method produces reliable, reproducible quantitative results within the defined concentration range while reducing analytical costs and sample preparation time. Although the method demonstrated acceptable validation parameters (described below), the lack of an isotopically labelled IS remains a limitation of the developed method. Its use would further enhance method robustness and analytical reliability and should be considered in future studies.

### 2.3. Method Validation

The developed method was validated, taking into account the guidelines of the International Conference on Harmonisation (ICH) and European Medicines Agency (EMA) [13,15]. Method validation included selectivity, linearity, lower limit of detection (LLOD), lower limit of quantification (LLOQ), recovery, matrix effect, accuracy (intra-day, inter-day), and precision (intra-day, inter-day). A limitation of the present study is the lack of stability assessment of the investigated drugs in saliva samples.

#### 2.3.1. Linearity

Calibration curves with six concentration points were created to assess linearity. Samples previously treated with the described SPE technique were analysed to generate the calibration curves. Table 3 displays the equations for the regression coefficients and calibration curves. Within the measured calibration range, the developed LC–MS/MS method was linear (r^2^ > 0.99) within the tested calibration range of 0.2–50 ng/mL (serum), 0.2–100 ng/mL (saliva), and 0.2–200 ng/mL (urine).

#### 2.3.2. Lower Limit of Detection (LLOD) and Lower Limit of Quantification (LLOQ)

The obtained LLOD values were 0.001 ng/mL for CAR and 0.003–0.008 ng/mL for LUR (Table 3). The LLOQ was 0.2 ng/mL for both drugs investigated in serum, saliva, and urine samples.

#### 2.3.3. Precision and Accuracy

The intra- and inter-assay validation data are presented in Table 4 and Table 5. The intra-day accuracy values ranged from 85.33 to 117.5% and from 80.83 to 113.33% for LUR and CAR, respectively, in biological samples. The inter-day accuracy values were in the range of 85.33–118.06% and 85.28–113.89% for LUR and CAR, respectively. Intra-day precision expressed as %CV ranged from 3.47 to 16.75% for LUR and from 3.26 to 14.85% for CAR. Inter-day precision expressed as %CV ranged from 3.63 to 17.68% and from 3.48 to 16.44% for serum and saliva samples, respectively. The findings demonstrate acceptable criteria: 85–115% (80–120% for LLOQ) for accuracy and ≤15% (≤20% for LLOQ) for precision.

#### 2.3.4. Recovery and Matrix Effect

The obtained recoveries (Table 6) were in the range of 89.4–98.11% for LUR and 95.22–99.8% for CAR in biological samples. The matrix effect (Table 6) ranged from 85.4 to 114.9% for LUR and from 86.48 to 113.8% for CAR in the investigated samples.

#### 2.3.5. Selectivity

**Figure 3:**
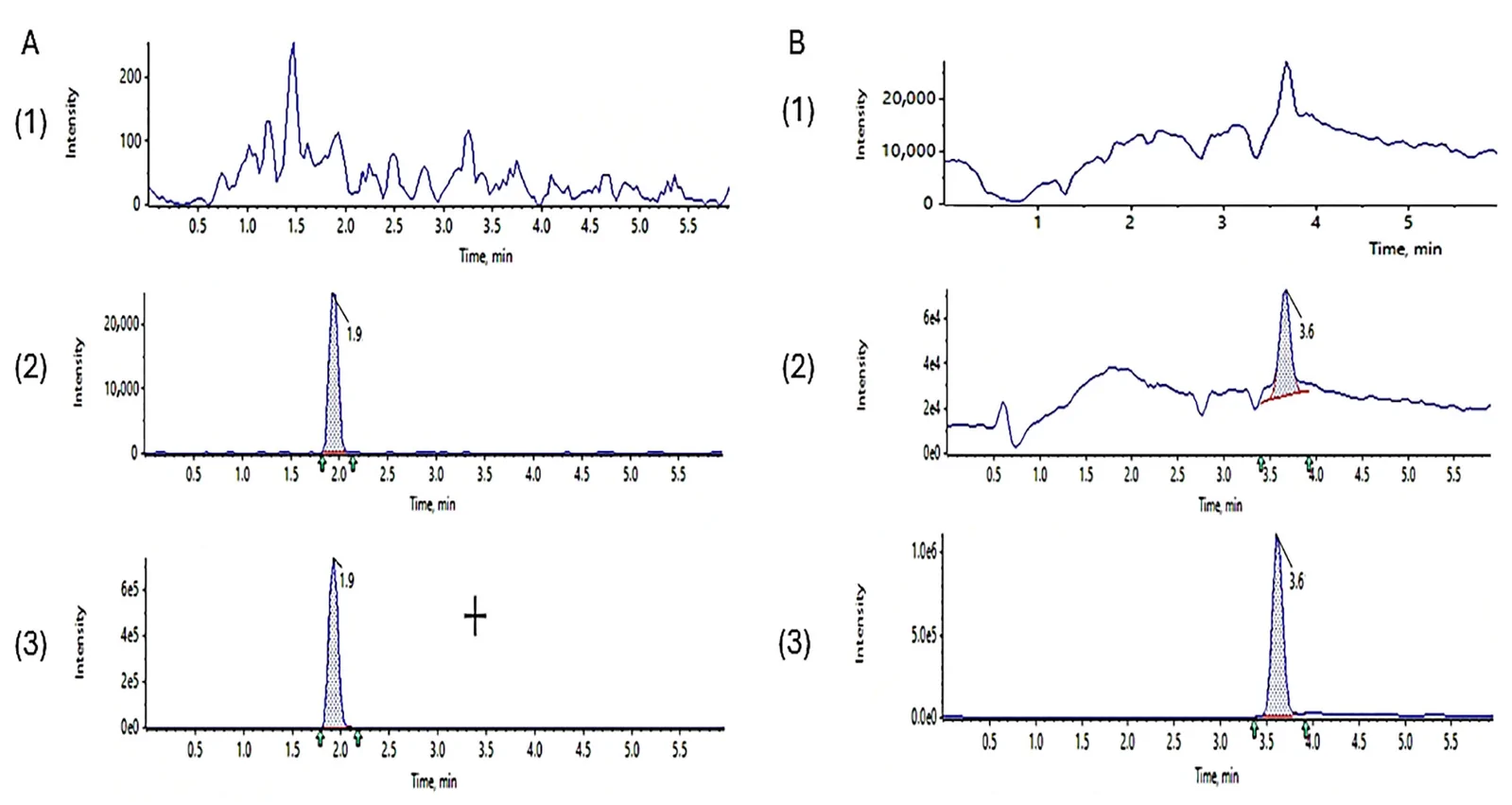
Chromatograms (MRM) obtained for human serum ((**A**)—CAR, (**B**)—LUR): blank (1), spiked with CAR and LUR at LLOQ level (2), and spiked with 10 ng/mL (3) of the investigated drugs. Arrows indicate the target analyte peak selected for analysis. Red lines denote the baseline used for peak integration.

**Figure 4:**
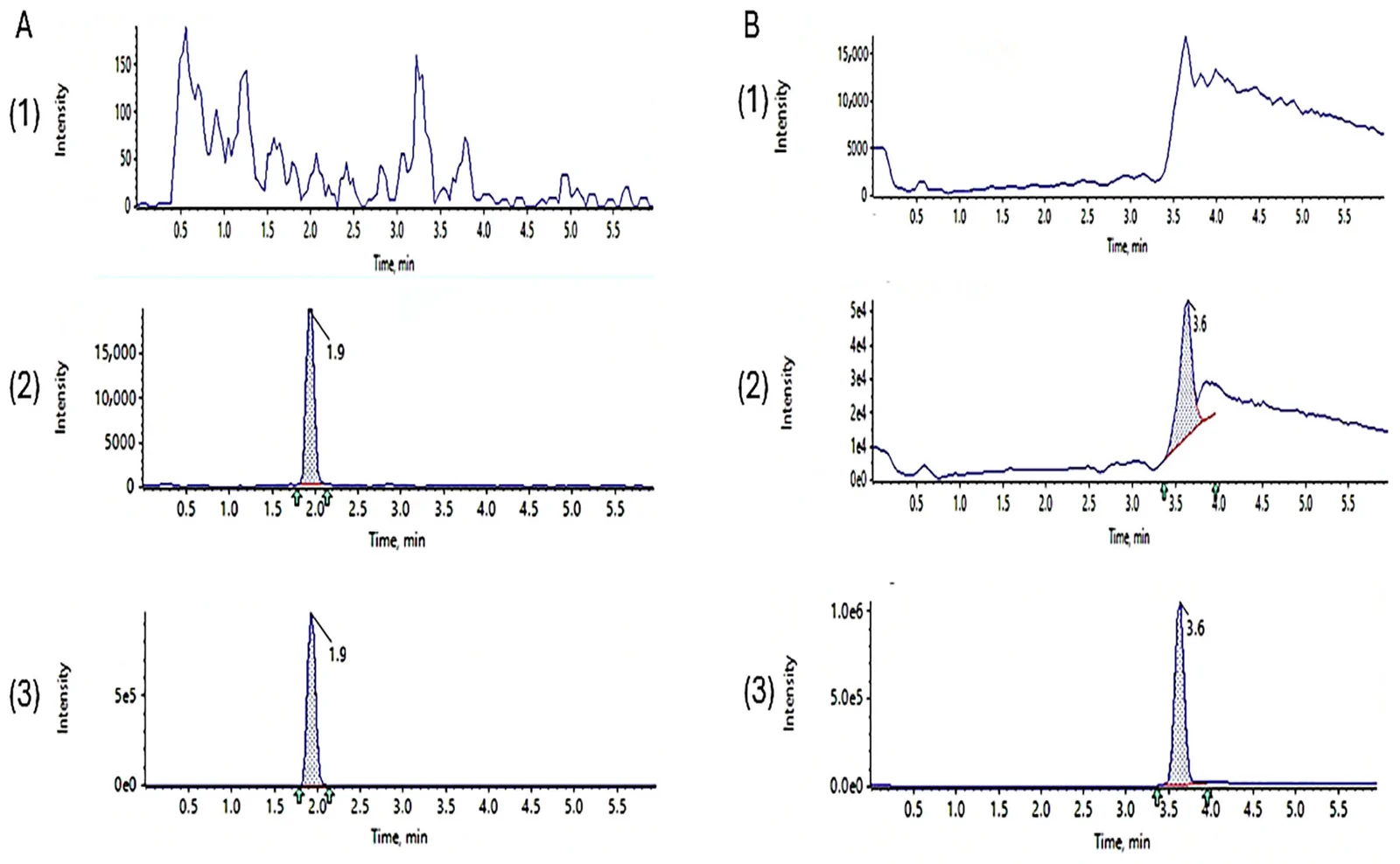
Chromatograms (MRM) obtained for human saliva ((**A**)—CAR, (**B**)—LUR): blank (1), spiked with CAR and LUR at LLOQ level (2), and spiked with 10 ng/mL (3) of the investigated drugs. Arrows indicate the target analyte peak selected for analysis. Red lines denote the baseline used for peak integration.

**Figure 5:**
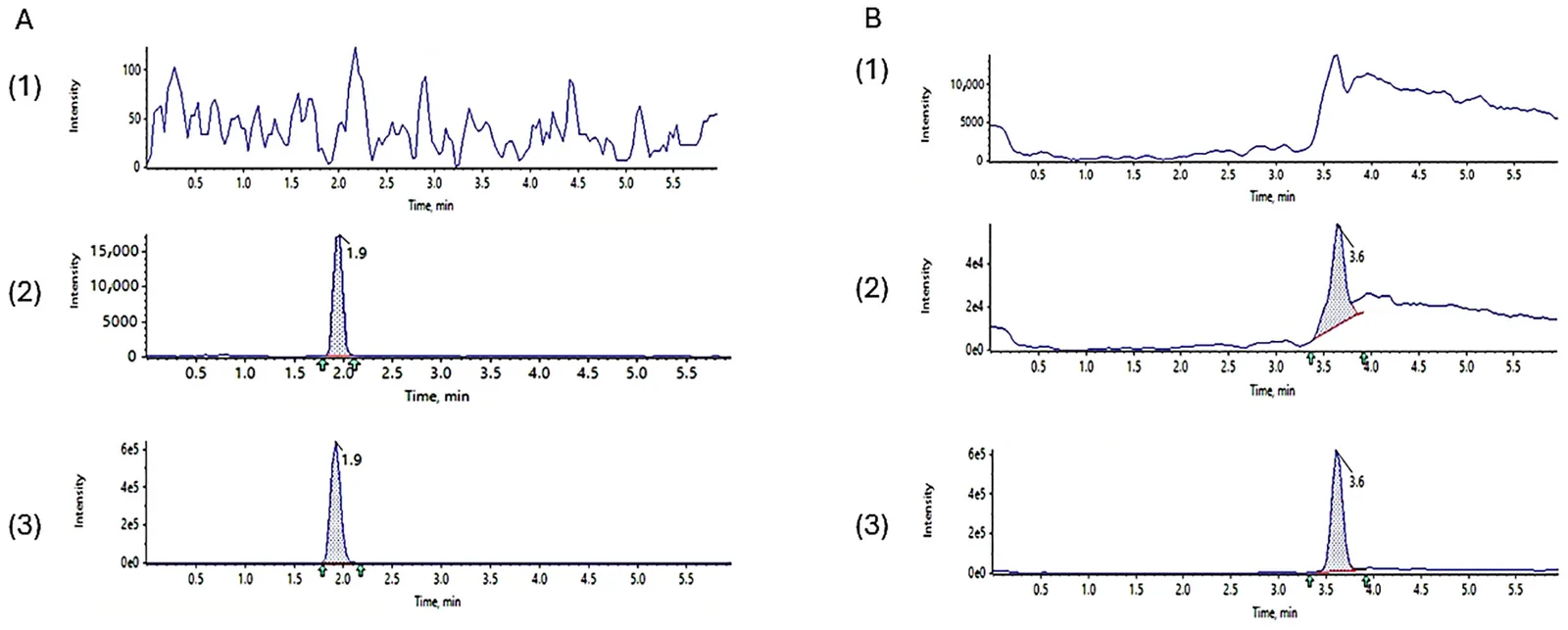
Chromatograms (MRM) obtained for human urine ((**A**)—CAR, (**B**)—LUR): blank (1), spiked with CAR and LUR at LLOQ level (2), and spiked with 10 ng/mL (3) of the investigated drugs. Arrows indicate the target analyte peak selected for analysis. Red lines denote the baseline used for peak integration.

An assessment of selectivity showed that there was no interference from matrix components in saliva, serum and urine samples that was significant for the quantification of LUR and CAR. Figure 3, Figure 4 and Figure 5 show chromatograms obtained for human serum, saliva and urine: blank, spiked with CAR and LUR at LLOQ level (0.2 ng/mL), and spiked with 10 ng/mL of the investigated drugs.

### 2.4. Application of HPLC-QTRAP-MS/MS Method for Determination of CAR and LUR in Serum, Saliva and Urine

**Figure 6:**
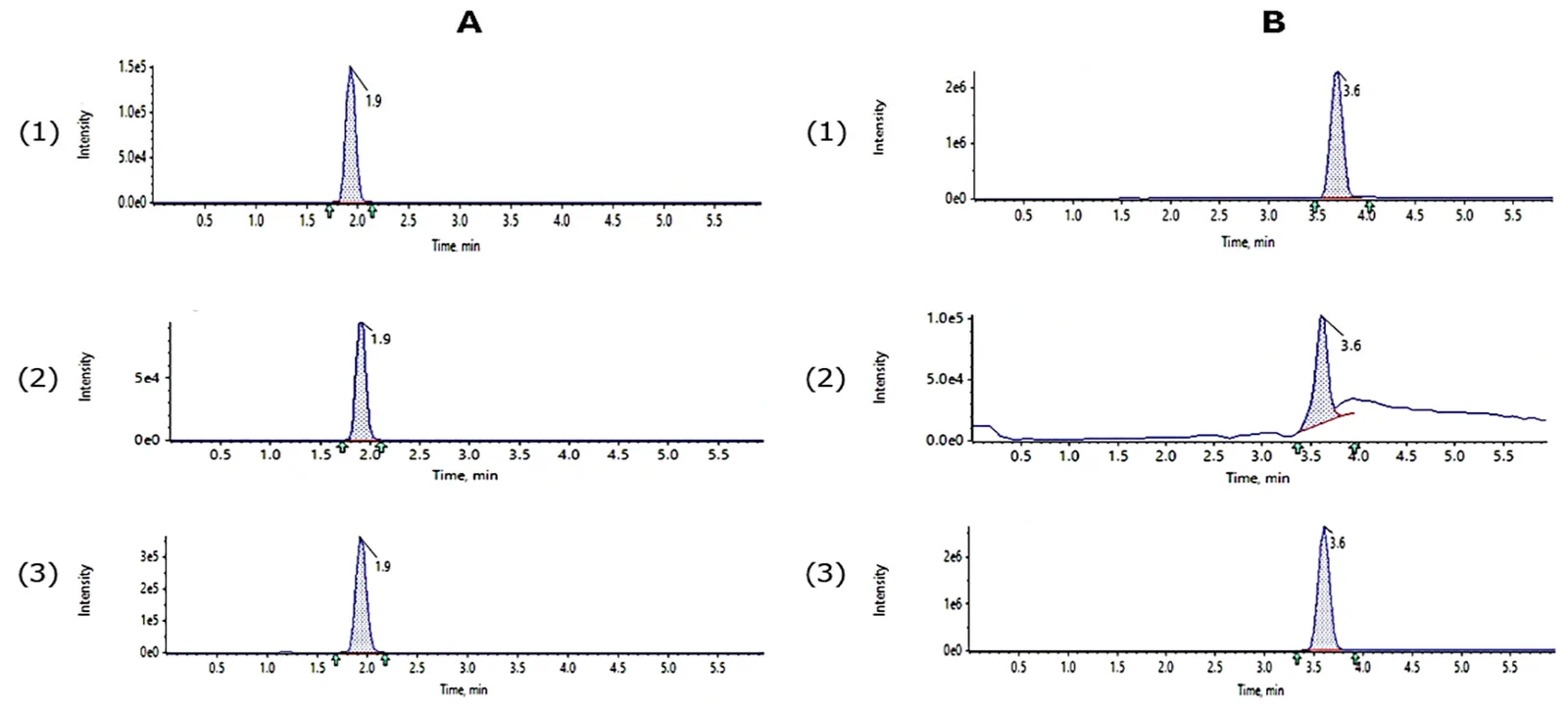
Examples of chromatograms (MRM) of CAR (**A**) and LUR (**B**) in human serum (1), saliva (2), and urine (3) obtained from patients treated with the investigated drugs. Arrows indicate the target analyte peak selected for analysis. Red lines denote the baseline used for peak integration.

The developed method has been successfully applied for the determination of CAR and LUR in biological samples from patients treated with these drugs. A sample of eight patients was analysed: four patients (men, aged 22–44 years) treated with CAR and four patients (two women, two men, aged 21–46 years) treated with LUR. In addition to the above-mentioned drugs, patients were taking other psychotropic drugs and medicines from other groups. Due to the small sample size and the varied treatment regimens, the effect of concomitant medications on the concentrations of the substances under investigation was not assessed. The results of the drug assays are shown in Table 7. Examples of obtained chromatograms are shown in Figure 6. The therapeutic reference range of LUR is 15–40 ng/mL and CAR is 10–20 ng/mL in serum/plasma [16]. The proposed analytical methods allow for monitoring the drug concentrations in biological samples. To date, the detection and quantification of the drugs under investigation have been carried out in serum/plasma and urine [5,10,11,17]. In this study, the investigated drugs were analysed in saliva for the first time. CAR concentrations ranged from 1.70 to 18.34, 2.10 to 36.48, and 0.63 to 6.96 in serum, urine and saliva, respectively. For LUR, the measured values ranged from 2.05 to 25.47, 9.57 to 148, and 0.21 to 0.67 in serum, urine and saliva, respectively. It should be noted that LUR concentrations in saliva were very low, close to the limit of quantification and, in two cases, below the LLOQ. This may pose a challenge in applying the method for drug analysis in saliva and its potential use in therapeutic drug monitoring. Considerable interindividual variability in drug concentrations was observed. For example, serum CAR concentrations at the same dose (3 mg) varied from 4.07 to 18.34 ng/mL. Similar between-subject differences were observed for LUR concentrations in the analysed biological samples. However, the small patient cohort does not permit reliable characterisation of population-level interindividual variability. This constitutes a limitation of this study.

A statistical analysis of the results was also performed (Tables S1–S3). As the presented research was a pilot study with a very small sample size, the statistical power is very limited. There is a statistically significant positive correlation between the CAR dose and serum concentration (ρ = 0.756, *p* = 0.049). Despite a fairly high correlation coefficient between the drug dose and concentrations in saliva and urine (ρ = 0.791 and 0.661, respectively), the sample size was too small to achieve statistical significance. A significant correlation was also found between the LUR dose and serum concentration (ρ = 0.754, *p* = 0.031). A fairly strong correlation was found between serum and saliva concentrations of CAR and LUR (ρ = 0.7 and 0.829, respectively), with statistical significance for LUR. Weak and moderately strong correlations were demonstrated between serum and urine concentrations of CUR and LUR, respectively. In both cases, there was no statistical significance.

Saliva offers an attractive alternative to samples such as serum and plasma. It is a non-invasive and safe method. Non-invasive sampling techniques have attracted considerable interest among scientists in recent years and may also be used in pharmacological research to enhance our understanding of drug metabolism and transport [18]. The results of the present study confirm the usefulness of the analytical method developed, including the feasibility of using saliva to determine CAR and LUR. However, no statistically significant correlation was found between serum and saliva CUR concentrations. For LUR, although statistical significance was observed (*p* = 0.042), the measured concentrations were close to the LLOQ, and in two samples, the levels were below the LLOQ. The low saliva-to-serum concentration ratio for LUR (0.01–0.06) may indicate limited penetration of LUR into saliva. A relatively high variability in saliva/serum ratios was observed for CAR (from 0.29 to 1.56). This finding may reflect interindividual pharmacokinetic variability, differences in salivary physiology, and protein binding. Due to the limited number of clinical samples, further studies involving a larger population are required to characterise the relationship between saliva and serum concentrations before saliva can be considered a reliable alternative for therapeutic drug monitoring. The present study is of a preliminary clinical nature.

## 3. Materials and Methods

### 3.1. Chemicals and Reagents

Hydrochloric acid, ammonium 25%, formic acid for LC-MS (98–100%), ammonium formate for LC-MS, acetonitrile for LC-MS, methanol for LC-MS, water for LC-MS and anhydrous sodium acetate were obtained from Merck (Darmstadt, Germany). Ammonium chloride was obtained from Chempur (Piekary Śląskie, Poland). Diethylamine was purchased from Sigma-Aldrich (Saint Louis, MO, USA). Standards of CAR and LUR were purchased from Gedeon Richter (Budapest, Hungary) and Aziende Chimiche Riunite Angelini Francesco (Rome, Italy), respectively.

### 3.2. HPLC-DAD Conditions

The investigated drugs were analysed using an LC-20AD Shimadzu (Shimadzu Corporation, Canby, OR, USA) liquid chromatograph equipped with a Shimadzu SPD-M20A detector set in the 200–800 nm range (Shimadzu Corporation, Canby, OR, USA), a thermostat CTO-10ASVP (Shimadzu Corporation, Canby, OR, USA), and a Rheodyne 20 µL injector. The eluent flow rate was 1.0 mL/min. The column oven temperature was set at 22 °C. The various stationary phases (Table 8) and eluents were used in chromatographic analysis. LabSolutions software version 5.71 (Shimadzu Corporation, Kyoto, Japan) was used for data acquisition and processing.

### 3.3. HPLC-MS/MS Conditions

The chromatographic analyses were carried out with the use of the UHPLC ExionLC AC system (SCIEX, Framingham, MA, USA), equipped with a hybrid triple-quadrupole linear ion trap mass spectrometer QTRAP 5500+ (SCIEX, Framingham, MA, USA). The ExionLC AC system included an ExionLC solvent valve, an ExionLC AC pump, an ExionLC AC autosampler, an ExionLC controller, an ExionLC AC column oven, and an ExionLC degasser. HPLC-MS/MS analyses were performed at a flow rate of 0.8 mL/min using a HPLC column (Synergi™ 2.5 µm Polar-RP 80 Å, 100 × 3 mm, Phenomenex, Torrance, CA, USA) maintained at 25 °C. Additionally, 5 mM formate buffer at pH 4.0 (A) and acetonitrile (B) were used as the mobile phase. The following solvent gradient was used: 0–4 min, 55–80% B; 4–5 min, 80% B; 5.1–6 min, 55% B. Injection volume was 5 μL. The QTRAP 5500+ was operated in positive mode electrospray ionisation (ESI+) using the following settings: curtain gas (CUR): 35 psi; collision gas (CAD): 9 psi; ion spray voltage (IS): 5500 V; source temperature (TEM): 500 °C; ion source gas 1 and 2: 60 psi. The declustering potential (DP), collision energy (CE), entrance potential (EP), collision cell exit potential (CXP) and mass transitions were optimised for each analyte (Table 2).

### 3.4. Biological Sample Collection

Biological samples (blood, saliva, urine) were collected from patients with mental illness who were being treated with antipsychotic medication and were admitted to Clinical Hospital No. 1 in Lublin, Poland. The study protocol was approved by the Bioethics Committee of the Medical University of Lublin (approval number: KE-0254/200/10/2022). Written informed consent was obtained from all participants before enrolment in the study. Patients participating in the study received LUR (*n* = 4) or CAR (*n* = 4), along with other antipsychotic and non-antipsychotic medications. Samples were collected at the same time, in a steady state, just before the administration of the next dose of the drug.

A Salivette kit (Sarstedt, Nümbrecht, Germany) was used to collect the saliva samples. The patient took the swab out of the Salivette and put it in the mouth and chewed to induce salivation for roughly sixty seconds. The swab with the saliva that had been absorbed was then put back into the Salivette. The Salivette was then centrifuged for two minutes at 1000× *g*, producing clear saliva. A sample was collected in the conical tube. Samples of saliva were kept at −20 °C until the extraction procedure.

Five millilitres of venous blood was drawn from patients receiving the investigated drugs. The collected blood was incubated for 30 to 40 min at room temperature (15 to 24 °C) until a clot formed. Following blood coagulation, the samples were centrifuged at 1500× *g* for 10 min. Before further analysis, the serum was separated and kept at −20 °C.

The urine (about 50 mL) collected in a plastic urine container was stored at a temperature of −20 °C.

### 3.5. Sample Pre-Treatment

Samples were prepared before chromatographic analysis by solid-phase extraction (SPE) using a SPE chamber—Baker SPE—12 G J.T. Baker (J.T. Baker, Philipsburg, NJ, USA) and Strata X-C cartridges (30 mg/mL, Phenomenex) or BAKERBONDTM SPE Octadecyl (C18) cartridges (100 mg/mL) (J.T. Baker, Phillipsburg, NJ, USA).

#### 3.5.1. Procedure 1

For the isolation of the investigated compounds from serum and saliva samples, SPE was performed using a Strata-X-C extraction column. For this purpose, the method developed by K. Wróblewski et al. [19] was modified. The 1 mL of sample was then diluted with 1 mL of 0.1 mol/L hydrochloric acid before the extraction procedure. Initially, the Strata-X-C extraction column was conditioned with 1 mL of methanol, followed by 1 mL of 0.1 mol/L hydrochloric acid solution. Next, the sample was introduced to the column at a flow rate of 1 mL/min, followed by washing with 1 mL of 0.1 mol/L hydrochloric acid solution and 1 mL of methanol. The column was dried under vacuum for 3 min, and the analysed compounds were eluted with a mixture of methanol and ammonia (prepared in a ratio of 95:5) twice with 1 mL. The eluate was evaporated to dryness, dissolved in 200 µL of methanol, and an appropriate aliquot was subjected to chromatographic analysis.

#### 3.5.2. Procedure 2

The extraction procedure was adopted from the method developed by Wróblewski et al. [20] and appropriately modified (concentrate the sample after SPE to 0.2 mL instead of 0.5 mL). Notably, 1mL of the urine sample was diluted with 1 mL of ammonium buffer at pH 8.6. Initially, C18 SPE columns were activated using 1 mL of methanol and then conditioned using 1 mL of a mixture containing redistilled water and ammonium buffer at pH 8.6 (5:1, *v*/*v*). The urine sample was then introduced to the SPE columns at a speed of 1 mL/min. The columns were prewashed with 1mL of a solution of MeOH and water (1:4, *v*/*v*) and then dried under vacuum for 3 min. Next, the extracted compounds were eluted twice with 1 mL of a solution of MeOH and glacial acetic acid (98:2, *v*/*v*). The sample was evaporated to dryness, dissolved in 200 µL of methanol, and an appropriate aliquot was subjected to chromatographic analysis.

### 3.6. Preparation of Stock Solution and Working Solutions

The stock-standard solutions of CAR and LUR at a concentration of 0.2 mg/mL were prepared in MeOH. The solutions were protected from light and stored at −20 °C. Before analysis, stock solutions were diluted in MeOH to create the working standard solutions.

### 3.7. Method Validation

The analytical method was validated using spiked human biological samples (serum, saliva, and urine), in accordance with International Conference on Harmonisation (ICH) and European Medicines Agency (EMA) guidelines [13,15]. The validation study included the evaluation of linearity, lower limit of detection (LLOD), lower limit of quantification (LLOQ), precision, accuracy, selectivity, extraction recovery, and matrix effect.

#### 3.7.1. Linearity

Linearity was assessed by preparing calibration curves, plotting the peak area ratios against the concentrations of CAR and LUR. Blank biological samples were spiked with suitable amounts of the drug standard. Samples were pre-treated by SPE and determined by the HPLC-QTRAP-MS/MS method. Calibration curves were constructed by analysing spiked samples at six concentrations, ranging from 0.2 to 50 ng/mL for serum, from 0.2 to 100 ng/mL for saliva, and from 0.2 to 200 ng/mL for urine samples. The calibration curve included a blank sample and non-zero samples, including the LLOQ.

#### 3.7.2. Lower Limit of Detection (LLOD) and Lower Limit of Quantification (LLOQ)

LOD was evaluated by the determination of the signal-to-noise ratio of 3:1. The LLOQ was calculated assuming that accuracy, precision, and repeatability were within 20% of the nominal values, and that the signal-to-noise ratio was no less than 10:1. The LLOQ was applied as the lowest calibration standard.

#### 3.7.3. Precision and Accuracy

Intra-day precision and accuracy were evaluated on the same day by analysing six replicates of quality control (QC) samples at four concentration levels—low QC (LLOQ), two mid-QCs, and high QC: 0.2 ng/mL, 10 ng/mL, 20 ng/mL and 30 ng/mL for serum samples; 0.2 ng/mL, 10 ng/mL, 30 ng/mL, and 90 ng/mL for saliva samples; 0.2 ng/mL, 10ng/mL, 50 ng/mL and 120 ng/mL for urine samples. The inter-day accuracy and precision were evaluated on three separate days by analysing QC samples at each concentration level.

#### 3.7.4. Extraction Recovery and Matrix Effects

Extraction recovery and matrix effects were calculated at three concentration levels based on the following formulas:

Matrix effect (%) = B/A × 100(1)

Recovery (%) = C/B × 100(2)

where A is the external solution peak area, B is the post-extraction sample peak area, and C is the extracted matrix peak area.

#### 3.7.5. Selectivity

Selectivity was assessed by analysing saliva, urine, and serum samples to investigate potential interference with the signals of LUR and CAR. The level of interference originating from endogenous sample components at the specific retention time of each analyte was assessed by comparing chromatograms of various batches of human biological samples with and without spiking with the investigated drugs.

### 3.8. Statistical Analysis

The assay results for the test drugs in biological samples from patients were subjected to statistical analysis. The Shapiro–Wilk test was used to determine whether the distributions were normal. A *p*-value > 0.05 was taken as indicating no basis to reject the hypothesis that the distribution was normal. Due to the small sample size and the failure of some of the analysed variables to meet the assumptions of normality, non-parametric methods were used. The relationships between the variables were assessed using Spearman’s rank correlation coefficient (ρ). Results below the LOQ were excluded from the correlation analyses. For each analysis, the number of available paired observations (n_pair_) was reported. The criterion for statistical significance was set at a *p*-value of less than 0.05.

## 4. Conclusions

Various HPLC-DAD chromatographic systems for the analysis of CAR and LUR were compared. Differences in retention, peak shapes, and system efficiency of the investigated drugs were obtained. For both drugs, the highest system efficiency was achieved across all tested mobile phases using the Polar RP column. The above stationary phases were applied to develop an HPLC-QTRAP-MS/MS method for the quantification of drugs in biological samples. The method was successfully applied to determine CAR and LUR. Quantification of the above drugs in saliva was performed for the first time. The assay’s high sensitivity, good precision, and accuracy make it a useful tool in clinical and toxicological analysis. Determination of CAR and LUR in saliva may be a potentially attractive alternative to drug quantification in serum or plasma because saliva collection is simple, non-invasive, and painless. However, further studies are needed on a larger sample size.

## Figures and Tables

**Table 1:** Retention time, asymmetry factor and theoretical plate number per meter obtained for CAR and LUR in various chromatographic systems.

Column	Mobile Phase	Drug	Retention Time	Asymmetry Factor	N/m
Hydro RP	50% MeCN, 20% acetic buffer pH = 3.5, water and 0.025 mol/L DEA	CAR	4.09	1.71	8350
		LUR	16.46	1.40	5700
	70% MeOH, 20% acetic buffer pH = 3.5, water and 0.025 mol/L DEA	CAR	4.59	1.31	14,100
		LUR	18.98	1.04	8100
	30% MeCN, 30% MeOH, 20% acetic buffer pH = 3.5, water and 0.025 mol/L DEA	CAR	5.63	1.32	21,600
		LUR	29.37	1.29	10,200
Phenyl-Hexyl	50% MeCN, 20% acetic buffer pH = 3.5, water and 0.025 mol/L DEA	CAR	2.66	1.04	27,100
		LUR	9.99	1.12	11,860
	70% MeOH, 20% acetic buffer pH = 3.5, water and 0.025 mol/L DEA	CAR	4.66	1.08	32,000
		LUR	19.49	1.03	9400
	30% MeCN, 30% MeOH, 20% acetic buffer pH = 3.5, water and 0.025 mol/L DEA	CAR	4.59	1.26	14,700
		LUR	18.96	1.12	9750
Polar RP	50% MeCN, 20% acetic buffer pH = 3.5, water and 0.025 mol/L DEA	CAR	4.50	1.40	33,650
		LUR	14.57	1.16	17,200
	70% MeOH, 20% acetic buffer pH = 3.5, water and 0.025 mol/L DEA	CAR	7.42	1.29	25,100
		LUR	42.63	1.16	10,700
	30% MeCN, 30% MeOH, 20% acetic buffer pH = 3.5, water and 0.025 mol/L DEA	CAR	6.49	1.20	39,550
		LUR	23.94	1.06	61,400
HILIC A	90% MeCN and 0.1 M ammonium formate	CAR	4.20	2.17	5250
		LUR	2.18	1.77	41,800
HILIC B	90% MeCN and 0.1 M ammonium formate	CAR	3.02	2.05	50,750
		LUR	2.01	1.60	7850
HILIC N	90% MeCN and 0.1 M ammonium formate	CAR	2.33	1.54	4350
		LUR	2.48	1.43	19,850
SCX	25% MeCN and phosphate buffer at pH 2.5	CAR	>120	-	-
		LUR	>120	-	-

**Table 2:** Chromatographic and MS/MS parameters of CAR and LUR.

Compound	Q1 Mass (Da)	Q3 Mass (Da)	DP (Volts)	EP (Volts)	CE (Volts)	CXP (Volts)	t_R_ (min)
CAR	427.17 *	382.03 *	90	10	40	10	1.9
		339.16	90	10	40	10	
LUR	493.19 *	166.11 *	100	10	60	10	3.6
		177.1	100	10	60	10	

* The MRM transitions selected for quantitative analysis.

**Table 3:** Parameters of calibration curves for quantitative analysis of LUR and CAR in serum, urine and saliva: calibration curves’ equations, concentration range, regression coefficients (r^2^), lower limit of detection (LLOD), and lower limit of quantification (LLOQ).

Drug	Sample Type	Regression Equation	Range (ng/mL)	r^2^	LLOD (ng/mL)	LLOQ (ng/mL)
LUR	Serum	y = 7.55130e5x + 2.72462e5	0.2–50	0.9950	0.008	0.2
	Urine	y = 4.56196e5x + 1.33624e5	0.2–200	0.9938	0.006	0.2
	Saliva	y = 9.44745e5x +1.85055e5	0.2–100	0.9982	0.003	0.2
CAR	Serum	y = 5.44204e5x +8.04012e4	0.2–50	0.9975	0.001	0.2
	Urine	y = 4.81881e5x − 6225.94740	0.2–200	0.9980	0.001	0.2
	Saliva	y = 5.06032e5x + 7.53788e4	0.2–100	0.9902	0.001	0.2

**Table 4:** Validation data: intra-day and inter-day accuracy and precision obtained for LUR.

Type of Sample	Concentration Added (ng/mL)	Intra-Day			Concentration Added (ng/mL)	Inter-Day		
		Concentration Found (ng/mL)	Accuracy (%)	Precision (%CV)		Concentration Found (ng/mL)	Accuracy (%)	Precision (%CV)
Serum	0.2	0.21	105	14.13	0.2	0.21	104.17	16.73
	10	11.28	112.83	9.05	10	11.13	111.28	10.24
	20	21.32	106.58	5.91	20	20.64	103.22	7.17
	30	27.43	91.44	7	30	26.7	89	7.27
Urine	0.2	0.18	90.83	11.76	0.2	0.19	93.06	12.52
	10	10.5	105	8.73	10	10.61	106.06	9.33
	50	54.03	108.07	7.06	50	53.08	106.17	7.59
	120	112.58	93.82	4.19	120	112.18	93.48	4.51
Saliva	0.2	0.24	117.5	16.75	0.2	0.24	118.06	17.68
	10	8.53	85.33	5.43	10	8.63	86.33	6.31
	30	28.52	95.06	4.74	30	28.1	93.67	5.67
	90	92.25	102.5	3.47	90	93.28	103.64	3.63

**Table 5:** Validation data: intra-day and inter-day accuracy and precision obtained for CAR.

Type of Sample	Concentration Added (ng/mL)	Intra-Day			Concentration Added (ng/mL)	Inter-Day		
		Concentration Found (ng/mL)	Accuracy (%)	Precision (%CV)		Concentration Found (ng/mL)	Accuracy (%)	Precision (%CV)
Serum	0.2	0.16	80.83	14.85	0.2	0.17	85.28	15.9
	10	8.8	88	6.82	10	8.76	87.56	7.06
	20	19.27	96.33	6.16	20	20.05	100.25	7.83
	30	31.85	106.17	4.43	30	29.5	98.33	6.65
Urine	0.2	0.23	113.33	11.73	0.2	0.23	113.89	12.47
	10	9.45	94.5	6.3	10	9.56	95.56	7.05
	50	53.03	106.07	6.05	50	52.49	104.98	7.08
	120	120.52	100.43	3.26	120	121.01	100.84	3.48
Saliva	0.2	0.16	81.67	12.65	0.2	0.2	100.56	16.44
	10	10.84	108.4	8.75	10	10.74	107.36	9.24
	30	32.77	109.22	6.32	30	30.83	102.76	7.46
	90	85.18	94.65	3.77	90	84.84	94.27	4.01

**Table 6:** Recovery and matrix effect obtained for CYT in serum and saliva samples.

Type of Sample	Concentration Added (ng/mL)	LUR				CAR			
		Recovery (%)	SD	Matrix Effect (%)	SD	Recovery (%)	SD	Matrix Effect (%)	SD
Serum	0.2	98.11	10.12	114.89	11.8	95.22	8.9	113.8	9.9
	10	96.55	7.5	88.14	8.1	96.30	8	95.07	8.3
	30	96.97	4.2	95.75	4.8	98.12	4.3	100.87	5
Saliva	0.2	94.41	11.1	113.64	12.1	96.12	9.8	113	10.12
	10	97.62	7.9	95.89	6.1	98.1	7	86.48	7.4
	90	95.03	3.9	104.29	4.05	99.8	3.9	93.67	4.6
Urine	0.2	89.4	9.6	85.4	9.1	95.9	9.3	111.2	9.2
	50	89.95	3	93. 38	4.84	99.2	4.1	96.77	3.2
	120	89.73	3.3	93.46	3.1	97.8	4	98.03	3.8

**Table 7:** Measured concentrations of CAR and LUR in serum, urine and saliva.

Drug	Patient No	Dose (mg)	Serum (ng/mL)	Urine (ng/mL)	Saliva (ng/mL)	Saliva/Serum Ratio
CAR	1	3.00	5.45	3.80	No collected	-
		4.50	5.85	10.24	6.23	1.06
	2	1.50	1.70	8.01	2.66	1.56
		4.50	10.17	36.48	6.96	0.68
	3	1.50	1.97	2.10	0.63	0.32
		3.00	4.07	3.41	1.16	0.29
	4	3.00	18.34	2.70	No collected	-
LUR	5	18.50	2.73	9.57	<LLOQ	-
		55.50	5.07	30.75	0.21	0.04
		74.00	13.68	119.80	0.24	0.02
	6	18.50	2.05	148.00	<LLOQ	-
	7	37.50	22.06	77.82	0.31	0.01
		55.50	21.61	58.39	0.29	0.01
		74.00	25.47	58.18	0.67	0.03
	8	18.50	4.72	47.69	0.28	0.06

**Table 8:** Tested chromatographic columns and their physicochemical properties.

Column	Functional Group	Length (mm)	Inner Diameter I.D. (mm)	Endcapped	Particle Size (µm)	Pore Size (Å)	Surface Area (m^2^/g)	Carbon Load (%)	Recommended pH Range
Synergi Hydro-RP	Octadecyl (C18)	150	4.6	Proprietary (polar group)	4	80	475	19	1.5–7.5
CSH Phenyl-Hexyl	Phenyl-Hexyl	150	4.6	Proprietary	5	130	185	15	1.0–11.0
Synergi Polar RP	Ether-linked phenyl	150	4.6	Proprietary (polar group)	4	80	475	11	1.5–7.0
Synergi Polar RP	Ether-linked phenyl	100	3	Proprietary (polar group)	2.5	100	475	11	1.5–7.0
ACE HILIC-A	Proprietary SIL	150	4.6	NO	5	100	300	-	2.0–7.0
ACE HILIC-N	Proprietary Polyhydroxy	150	4.6	NO	5	100	300	7	2.0–7.0
Luna SCX	Benzene Sulfonic Acid	150	4.6	NO	5	100	400	0.55 Sulphur Load	2.0–7.0

## Data Availability

Data are available from the corresponding author upon reasonable request.

## Supplementary Materials

The following supporting information can be downloaded at: https://www.mdpi.com/article/10.3390/ijms27167090/s1.
