# Supplementary material for: From Chromatographic Optimisation to Bioanalysis: HPLC System Comparison and HPLC–QTRAP–MS/MS Determination of Cariprazine and Lurasidone in Human Serum, Urine, and Saliva
Source: Int J Mol Sci. 2026 Aug 7;27(16):7090. doi: 10.3390/ijms27167090 (PMC13513814; doi:10.3390/ijms27167090)
Supplement: Supplementary file 1 [file ijms-27-07090-s001.zip › ijms-4340956-supplementary.pdf]

Table S1. Assessment of distribution normality (Shapiro–Wilk test).

Variable	Drug			
	CAR		LUR	
	Result	p-value	Result	p-value
Drug concentration in serum	normal distribution	0.103	normal distribution	0.092
Drug concentration in urine	non-normal distribution	0.0013	normal distribution	0.635
Drug concentration in saliva	normal distribution	0.273	non-normal distribution	0.006

Table S2. Exploratory analysis of the relationship between administered dose and drug concentrations in serum, saliva, and urine samples.

Drug	Relationship	n_{pair}	ϱ (Spearman)	p-value
CAR	Dose ↔ Serum drug concentration	7	0.756	0.049
	Dose ↔ Saliva drug concentration	5	0.791	0.111
	Dose ↔ Urine drug concentration	7	0.661	0.106
LUR	Dose ↔ Serum drug concentration	8	0.754	0.031
	Dose ↔ Saliva drug concentration	6	0.059	0.912
	Dose ↔ Urine drug concentration	8	0.173	0.682

n_{pair} -the number of available paired observations; ϱ (Spearman) - Spearman's rank correlation coefficient; p-value - significance level of the correlation test. The criterion for statistical significance was set at a p-value of less than 0.05.

Table S3. Assessment of the relationship between drug concentration in serum and saliva or urine.

**Drug**	**Relationship**	**n_{pair}**	**ϱ (Spearman)**	**p-value**
CAR	Serum ↔ Saliva	5	0.70	0.188
	Serum ↔ Urine	7	0.21	0.645
LUR	Serum ↔ Saliva	6	0.829	0.042
	Serum ↔ Urine	8	0.643	0.119

n_{pair} -the number of available paired observations; ϱ (Spearman) - Spearman's rank correlation coefficient; p-value - significance level of the correlation test. The criterion for statistical significance was set at a p-value of less than 0.05.
